# Privacy and Human-AI Relationships

**DOI:** 10.1007/s13347-025-00978-2

**Published:** 2025-10-18

**Authors:** Christopher Register, Maryam Ali Khan, Alberto Giubilini, Brian David Earp, Julian Savulescu

**Affiliations:** 1 Uehiro Oxford Institute, University of Oxford, Suite 1 Littlegate House, St Ebbe’s Street, Oxford OX1 1PT, England; 2 Centre for Biomedical Ethics, Yong Loo Lin School of Medicine, National University of Singapore, Blk MD 11, 10 Medical Dr, #02-03, Singapore 117597, Singapore

**Keywords:** Privacy, Artificial intelligence, Human-AI relationships, Relational moral psychology

## Abstract

Artificial intelligence (AI) agents such as chatbots and personal AI assistants are increasingly popular. These technologies raise new privacy concerns beyond those posed by other AI systems or information technologies. For example, anthropomorphic features of AI chatbots may invite users to disclose more information with these systems than they would otherwise, especially when users interact with chatbots in relationship-like ways. In this paper, we aim to develop a framework for assessing the distinctive privacy ramifications of AI agents, especially as humans begin to interact with them in relationship-like ways. In particular, we draw from prominent theories of privacy and results from human relational psychology to better understand how AI agents may affect human behavior and the flow of personal information. We then assess how these effects could bear on eight distinct values of privacy, such as autonomy, the value of forming and maintaining relationships, security from harm, and more.

## 1 Introduction

As artificial intelligence (AI) applications become entwined with everyday life, questions about privacy grow more pressing. Contemporary discussions of privacy in digital contexts often concentrate on data privacy and the hazards of unrestrained data collection (e.g., Susser et al., 2019; Véliz, 2020; Zuboff, 2019), and more recently on how AI applications affect information security (e.g., Blease, 2024; Li et al., 2023; Novelli et al., 2024; Williamson & Prybutok, 2024). Yet AI systems in the form of interactive AI *agents* bring distinctive privacy challenges, especially as humans increasingly form quasi-social or relationship-like engagements with these systems. In some scenarios, AI products already project humanlike qualities—consider conversational chatbots such as Replika—which invites users to treat them not merely as tools but as potential companions or confidants.

It is tempting to assume that the privacy ramifications of AI agents fit seamlessly into traditional debates about surveillance or data protection. Indeed, photography, CCTV, and large-scale webtracking have all sparked similar controversies in the past. But AI agents may be unique because humans readily slip into reciprocal, quasi-relationships with systems that exhibit anthropomorphic cues, such as fluent conversation or lifelike avatars. (We use ‘quasi-relationships’ to refer to patterns of human-AI interaction that mimic human relationships in some ways, even if the AI system doesn’t count as a real companion. We encourage the reader to be wary of how anthropomorphic language may be misleading (Floridi & Nobre, 2024; Salles et al., 2020).) Some emerging empirical literature suggests people may even share more personal details with these systems than they might with other humans or less sophisticated machines (Laban et al., 2023). Moreover, anthropomorphized AI can shape not just how people disclose information but also how they feel about that disclosure, whether it fosters comfort and trust or, conversely, leads to discomfort and inhibition (Schneeberger et al., 2019).

These developments raise the question: how, exactly, does an ongoing human-AI relationship differ from an isolated human-AI interaction, and why might this matter for privacy? Interacting once with a chatbot to handle a basic task may be akin to using any new digital tool, posing no radical privacy threat beyond more mundane data-collection. By contrast, forming a recurring quasi-relationship with an AI agent can elicit deeper disclosure, as the user feels a sense of trust or even empathy toward the system—feelings that, in turn, the AI may be specifically designed to encourage. At the same time, AI agents differ from human confidants in that they can store and analyze vast quantities of information indefinitely. Third parties, such as technology companies, may also have access to the data that AI agents elicit from users. Thus, beyond the familiar risks of data privacy breaches or of confiding in other people, human-AI relationships involve AI information processing with a human face—a wolf in sheep’s clothing.

The aim of this paper is to develop a framework for assessing how AI agents, in connection with human relational psychology, may diminish or reshape privacy. We take a broad perspective on privacy, integrating multiple theoretical traditions including access- and control-based accounts as well as the theory of contextual integrity (Nissenbaum, 2010). While debates about privacy usually treat human observers (e.g., a nosy neighbor) or institutions (e.g., corporations) as the primary threats, the possibility that AI systems themselves might function like observers with inferential or interpretive capacities suggests new ethical terrain. And, even if AI systems will not soon be conscious or cannot ever be conscious, they may still collect and infer personal information in ways that affect people’s autonomy, relationships, security, and more.

The framework suggests that AI agents present two threats to privacy: first, AI agents may directly diminish privacy by observing and acquiring new kinds of information about humans in a wider range of social settings; second, AI agents may indirectly diminish privacy by making this information available to third-parties, including other humans or institutions. The threats posed by AI agents are distinctive because AI agents diminish privacy to a greater degree than other technologies and, crucially, because AI agents will acquire information through quasi-social mechanisms. Because AI agents have a human face, so to speak, they will be in a position to acquire information about humans by leveraging their social position or human relational psychology to increase willing disclosure. The social and anthropomorphic features of AI agents then interact with other properties of AI systems to exacerbate threats to privacy, for example by exposing this new information to third-parties or by amplifying the potency of existing statistical inference. Thus, while human relational psychology and AI anthropomorphism are key difference-makers in generating the distinctive privacy impacts of AI agents, other more mundane features of AI systems take on increased significance in their wake.

The paper is organized as follows. In section 2, we discuss the nature and value of privacy which will form the foundation of our framework, drawing key insights from access, control, and contextual integrity theories. Section 3 then explains why human relational psychology is crucial for understanding how people interact with AI agents and how that affects the flow of information. In section 4, we examine how these patterns of interaction change privacy through the lenses of access, control, and contextual integrity. The result is a framework designed to guide the evaluation of the privacy impacts of AI agents informed by empirical psychology. Finally, in section 5, we draw out preliminary assessments from the framework, emphasizing the privacy risks that are introduced by the proliferation of AI agents. We do not draw conclusions about the all-things-considered goodness or badness of AI agents’ impact on privacy; our goal is to use the framework to assess how AI agents may affect each of eight values of privacy.

## 2 Insights from the literature on privacy

Our framework draws from three prominent theories of information privacy to assess the privacy impact of AI agents. Our framework provides two contributions: first, the framework integrates three theories information privacy, showing how they are complementary and how they emphasize distinct values of privacy. Second, the framework shows how properties of AI systems and human relational psychology interact to influence the flow of information in ethically significant ways. Taken as a whole, the framework aims to enable the comprehensive evaluation of how AI agents impact information privacy. In this section, we integrate access, control, and contextual integrity theories of information privacy. Before explaining these theories in more detail, we briefly situate the framework within broader debates about privacy.

Our focus is on information privacy, but there are other concepts of privacy. A concept of privacy that has been important in the legal domain is *decisional privacy*, which consists in freedom from interference in personal matters, such as the government’s involvement in who one marries or whether one may pursue an abortion (Griswold, 1965; Roe, 1973). Another traditional notion of privacy is *locational privacy*, which concerns privacy in a particular location, such as one’s own home (Roessler, 2005). We will set decisional and locational privacy aside and instead focus on information privacy because we take it to be more immediately affected by AI systems. In general, information privacy concerns privacy of personal information. As we conceive of it, information privacy concerns the *flow* of personal information, which provides a unifying conceptual ground for discussing three dimensions of access, control, and contextual integrity. Hereafter, ‘privacy’ refers specifically to information privacy.

A further caveat is that while we believe there is an important and distinctive notion of group privacy, we will limit our attention to individual privacy. A notion of group privacy requires additional resources and attention that we cannot pursue here, though this is a worthwhile avenue for future development (Floridi, 2016; Mittelstadt, 2017).

Legal and moral discussions sometimes focus on privacy infringements or violations. As these terms are typically understood, infringements and violations presuppose a right to privacy and can only be committed by moral agents. We will not discuss whether there is a right to privacy, whether AI companies or employees have privacy duties, or whether AI systems count as moral agents, nor will we presuppose any answers to these questions. Instead, our focus is only on the effects that AI systems may have, so we will instead talk about what *diminishes* privacy (cf. Macnish, 2020). Assessing how AI systems may diminish privacy is important regardless of rights and responsibilities, and this initial assessment is important for any eventual understanding of what rights and responsibilities there may be.

The three most prominent approaches theorize privacy in terms of access, control, and contextual integrity. Access is the acquisition of personal information by another party. Control concerns a person’s control over the transmission and usage of their information. Contextual integrity, as theorized in Nissenbaum (2010), concerns the flow of information within social contexts. Each of these conceptions has to do with the actual or potential flow of personal information, so we treat this broad notion as a unifying conception and discuss each of the three approaches as highlighting distinct aspects of this broader notion of privacy. In our view, each of these approaches captures elements of the flow of personal information that are valuable to individuals or to society. So, while our framework knits together distinct theories under a unified theme, the framework also espouses a highly pluralistic conception of the value of privacy.

Thus, the framework we offer marks both an ecumenical synthesis of existing theories of personal information privacy and a modest departure from each of those theories. For example, Solove (2008) endorses a pluralistic conception of the value of privacy and of privacy itself, while we instead emphasize a more unified conception of privacy—at least with respect to information privacy—that underwrites the value pluralism. While some authors criticize accounts based on access (e.g., Westin, 1967), control (e.g., Parent, 1983), or contextual integrity (e.g., Solove, 2008), we do not find it fruitful to abandon any of these conceptions. Instead, we think the most powerful framework is one that draws from each of them.

In what follows, we are primarily interested in information flow to and from AI systems of a particular type–what are called *AI agents*–because these systems are becoming especially prevalent and raise distinctive privacy concerns. AI agents are task-oriented AI products that interact with users and are able to act in the world, such as through conversation, via the internet, or through physical devices. So, our focus will be on how the use of AI agents in particular may diminish privacy. While the diminishment of privacy is not always bad or harmful, we will emphasize how privacy diminishment can be bad or harmful in more and less direct ways.

### 2.1 Privacy as limits on access

Some theorists hold that privacy consists in there being limits on access to one’s information or self (Thomson 1975, Gavison, 1980). Access can be conceived in terms of physical access, such as intrusion in private spaces, or informational access. Both may be important, but we will focus on informational access, whether through observation, monitoring, or inference. In the broadest sense, access includes being visually perceived, being recorded via CCTV cameras, having internet activity tracked, or observation through other surveillance mechanisms.

On this view, privacy norms serve to protect individuals from certain kinds of access. These norms may be justified by the value for individuals of not having their personal information accessed or known by others. The state of not being accessed may be finally valuable (i.e., an end in itself), such as if there is sanctity of a certain private domain of an individual’s life or if the individual has an interest in how they are presented to or interpreted by others (which is a matter of what information is accessed by others). It may also be instrumentally valuable, due to a further value in not suffering the downstream consequences of access, such as embarrassment, social approbation, or political oppression.

Arguably, only some kinds of information acquisition are relevant to the value of privacy as an end, such as observation by another human (Elliott & Soifer, 2022; Macnish, 2020; Soifer & Elliott, 2014). In contrast, *merely* being recorded by a CCTV camera or a webtracking bot does not itself affect such final values and instead can only bear on the instrumental values of privacy, such as if the camera footage or tracking data is used by an institution. Macnish (2020) even goes so far as to argue that only the former sort of information acquisition is truly a matter of privacy and hence that acquisition by, e.g., CCTV cameras does not in itself count as access. Since this paper addresses both instrumental and final privacy values, we are interested in both the broader and narrower notions of access, which we will call ‘weak access’ and ‘strong access’ respectively. Weak access consists in the acquisition of another’s personal information. Strong access consists in that information being somehow perceived or understood by another subject. Each kind of access diminishes privacy, albeit in ways that matter for different privacy values.

There are substantive questions about what the capacity for strong access requires in general and whether AI agents could possess it, as we will briefly discuss in section 4 (see also [OMITTED FOR BLIND REVIEW] for a lengthier discussion). If AI agents are capable of strong access, then they can directly diminish privacy and its final values even without human involvement. Call this *direct strong access*. If AI agents are themselves only capable of weak access, then they can still diminish privacy in two importantly different ways. First, AI agents may indirectly enable strong access by other humans, who then diminish privacy and its final values. Call this *indirect strong access*. Or, weak access by AI agents may affect the instrumental values of privacy, such as if an algorithm uses personal information to deny a job or mortgage application even when no humans are involved in the process.

### 2.2 Privacy as control

Privacy has also been conceptualized as a kind of *control* over access (in any form) to one’s information. Norms protecting an individual’s control over their personal information can also be justified by appeal to values. A value of privacy that is highlighted on control-based theories is the promotion of individual autonomy (Cohen, 2002; Decew, 1997; Westin, 1967). To the extent that one’s control over access is constrained, one’s autonomy is also limited. For the purposes of this paper, we will understand autonomy as control over important aspects of one’s own life. If autonomy in this sense is valuable in itself—as we assume it is—then reduced control over access to one’s personal information is disvaluable.

Control is also valuable because of the role it plays in self-presentation and relationship formation, each of which are often quite important to people (Marmor, 2015; Rachels, 1975; Shoemaker, 2010; Soifer & Elliott, 2014). Being able to control what information people have about you is important in influencing how they evaluate you. Self-presentation may be something we care about in itself, and it may also be something we care about because it enables the formation and maintenance of a diversity of desired relationships.

To the extent that AI systems can or do access individuals and their information, or facilitate others’ access, in ways the individuals cannot control, those systems will reduce privacy in this sense. Some argue that current AI systems are not especially harmful in these specific respects, despite the prevalence of large-scale data monitoring (Elliott & Soifer, 2022). We largely agree with this diagnosis in the context of non-anthropomorphic, non-interactive AI systems. However, as AI systems, especially in the form of AI agents, become more human-like and interactive in their speech, appearance, or behavior, we expect that they will have greater impact on autonomy, self-presentation, or relationship formation. We assess these effects in greater detail in section 5.

Control is also valuable because it can ensure several downstream safeguards, such as freedom from tyranny or targeted harassment, which promotes both individual security and the public interest in a free, democratic society (Goold, 2010; Stahl, 2020; Véliz, 2020).

### 2.3 Privacy as contextually appropriate information flow

Another prominent theory of privacy, and one that has been applied specifically to analyze the privacy impact of new technologies including AI, holds that privacy consists in contextually appropriate information flow (Brause & Blank, 2023; Gabriel et al., 2024; Nissenbaum, 2010, 2019). When information flow is contextually appropriate, it preserves *contextual integrity*. On this view, privacy norms specify when information should or should not be shared, and those norms depend on various parameters and may vary across contexts.

Contexts are structured institutional or social settings that involve roles, relationships, and norms, such as the patient-provider relationships in a healthcare setting. Contextual privacy norms are characterized by five parameters: the person whose information is involved (the data subject), the sender of information, the receiver of information, the information type, and the conventional transmission rule. For example, if a doctor shares my healthcare information with a stranger, then I am the data subject, the doctor is the sender, the stranger is the receiver, the information type is personal healthcare information, and the conventional transmission rule is something like *patient-provider confidentiality as encoded in healthcare information privacy laws*. This act of transmission would violate the transmission rule, constituting a breach of contextual integrity. On the contextual integrity theory of privacy, this is a breach of privacy. Box 1 summarizes the key elements of the theory of contextual integrity.

There are two key benefits of the theory of contextual integrity for our purposes. First, the theory highlights the contextual parameters along which privacy norms can vary. This is important because AI agents and the systems in which they are embedded introduce contextual changes to the flow of information, such as by introducing new senders or receivers of information or new information types, and may even change the shape of contexts themselves. The theory of contextual integrity draws our attention to these privacy-relevant effects.

Second, moving from the descriptive to the normative, the theory of contextual integrity provides a method for evaluating new information flow. Nissenbaum (2010, p. 181-2) describes the *augmented decision heuristic*: a method for identifying and ultimately evaluating changes to the integrity of contexts. In broad strokes, the augmented decision heuristic identifies transgressions of privacy norms in a context and then assesses how those transgressions affect contextual ends as well as other values, including individual interests and more general social or political ends (cf. Nissenbaum, 2019). Contextual ends include, for example, patient wellbeing in the healthcare context or student edification in a pedagogical context. Social and political ends may include public safety or the development of a competent electorate. If the balance of ends speaks against a transgression, then the augmented decision heuristic recommends against it.

The introduction of AI systems in society, and of human-AI relationships more specifically, changes the flow of information in important ways. These changes may transgress privacy norms, and the augmented decision heuristic helps us assess the transgressions. In principle, information flows may be altered by AI systems in multiple distinct ways:

1. AI systems may collect new types of information.
2. AI systems may involve third-party receivers.
3. AI systems may be new kinds of receivers, having new properties or roles.
4. AI systems may follow new transmission rules, perhaps even changing the conventionally established rule.

Each of these changes would disrupt contextual integrity, and each change could be assessed for how it promotes or frustrates contextual, individual, or social and political ends. We will discuss these changes in section 4 and assess them in section 5.

### 2.4 A taxonomy of values promoted by privacy

Privacy promotes many distinct values. Each conception of privacy discussed so far reveals some of these values. Access is valuable in part because it affects how one is interpreted. Control is valuable in part because autonomy is valuable. Contextual integrity secures various contextual values such as patient wellbeing. In Table 1 we briefly provide a more comprehensive list of values promoted by privacy and privacy norms. Our taxonomy of values is inspired by many works in the literature on privacy but does not strictly adhere to any particular theory (though see Solove, 2008 for a helpful review). These values will ultimately underpin our normative assessment of the ethical impact of AI agents on privacy in section 5.

## 3 AI Agents and Human Relational Psychology

To better understand how AI agents may affect privacy, both currently and in the near future, we must understand how AI systems function and how they affect human psychology. Privacy and privacy values are susceptible to how AI systems share information, influence human disclosure of information, and affect human feelings. Our primary goal in this section is rather modest: we aim to show that a better understanding of human relational psychology, especially in the context of human-AI interactions and relationships, is crucial for understanding the privacy effects of AI agents. Our secondary goal is to show that properties of AI agents interact with human psychology to pose two distinctive threats to privacy: first, the direct diminishment of privacy by increased acquisition of information by AI agents; second, the indirect diminishment of privacy by making this information available to thirdparties, including other humans or institutions. To establish these claims, we will discuss properties of the current generation of AI agents and recent findings about how humans interact with AI systems of various kinds.

Regarding our primary goal, it is crucial to note that theories of human-AI interaction are extensive and evolving. While a thorough review of existing theories is beyond the scope of our paper, we draw attention to two emerging themes. The first theme is that humans interact with AI in complex ways and treat AI systems according to human social patterns only sometimes and only in some respects (Earp et al., 2025; Gambino et al., 2020; Hortensius & Cross, 2018; Nass & Moon, 2000; Reinecke et al., 2025). A second theme, emerging from various theories of interpersonal relationships (Fox & Gambino, 2021; Taylor & Altman, 1987) and human-AI relational norms theory (Earp et al., 2025) is that human-AI interactions are dynamic and bidirectional, evolving as humans develop ongoing relationships with AI systems.

More and more, users are interacting with AI systems not merely as task-oriented tools, but as agents with which we have quasi-social interactions and may even form quasi-relationships (Manzini et al., 2024; Pentina et al., 2023). AI agents (and the larger systems in which they are embedded) have several properties that complicate the privacy landscape. We discuss six such properties here, summarized in Box 2.

The genuinely distinctive property of AI agents is their anthropomorphic features. This property sets AI agents apart from other information technologies because it brings human relational psychology into play. The other properties described in Box 2 are included because they interact with anthropomorphic features to exacerbate the effects on privacy. The overarching logic of the list is reflected in the order they are given: the earlier properties influence how information flows to AI agents, whereas later properties concern how information flows from AI agents. The list aims to capture the most important properties as described in the recent literature. Even so, this list should be understood as tentative given the continual and rapid evolution of AI technologies.

Recent results in human psychology also reveal patterns that, together with these six properties of AI agents, appear likely to influence the landscape of privacy in human-AI interactions. We draw attention to four such patterns, summarized in Box 3.

These four patterns interact in complex ways with each other and with the features of AI agents. We will discuss some of these interactions and draw out tentative predictions that will be relevant for the impact on privacy. Each of these patterns are brought into play because AI agents exhibit some degree of anthropomorphism, but the effects are magnified due to other properties of AI agents.

Privacy fatigue is a phenomenon where people tend to become overwhelmed by the demands of managing their personal privacy, either emotionally or cognitively (Choi et al., 2018; Oviedo-Trespalacios et al., 2023; Yang et al., 2023). Privacy fatigue has been studied in the context of social media interactions and the use of AI agents like ChatGPT (Chung & Kwon, 2025; Shao et al., 2022; Shim et al., 2024). One general effect of privacy fatigue is that, as people face increased privacy fatigue, their willingness and ability to withhold information decreases. As it becomes harder to control and monitor personal information, people often let down their guard and may disclose more information than they otherwise would. In the context of AI agents, privacy fatigue may then accelerate disclosure. Several features of AI agents mentioned in Box 2 make it increasingly difficult to track and manage personal privacy while interacting with these agents. The need to keep in mind unpredictable behavior, increased informational capacities, the difficulty of deleting information that AI models have been trained on, and the presence of lurking third parties increases the cognitive demands of managing personal privacy and heighten the risks of disclosing personal information. Instead of raising their defenses, users may *lower* their defenses in response, increasing their information disclosure. Further, anthropomorphic features may elicit emotions or and social judgments that impose additional cognitive demands on users (Shim et al., 2024). Compounding cognitive demands exacerbate the effects of privacy fatigue, which becomes especially potent given the other effects of anthropomorphic features.

The human tendency to anthropomorphize AI agents is especially strong given their anthropomorphic features. This tendency may contribute to downstream privacy risks in complex ways. Humans tend to anthropomorphize AI agents, such as by attributing consciousness or feelings to them (Colombatto & Fleming, 2024; Fetterolf & Hertog, 2023; Guingrich & Graziano, 2023) and such tendencies will likely increase as AI agents become more anthropomorphic (Geiselmann et al., 2023). This has facilitated the formation of relationships between AI and users (Brandtzaeg et al., 2022; Pentina et al., 2023). Empirical work suggests that, as humans interact in more frequent and richer ways with AI agents, this facilitates intensified information disclosure. For example, in a mixed-methods study of Replika users, anthropomorphization and perceived authenticity (e.g., whether the AI sounds scripted or like an independent, real mind) facilitated reciprocal information disclosure and relationship development (Pentina et al., 2023).

Thus, anthropomorphization contributes to a second pattern in human-AI relational psychology: the formation of relationship patterns, such as reciprocity and other feelings or dispositions, including comfort and a sense of rapport. We use ‘reciprocity’ to refer to conversational responsiveness and mutual disclosure. ‘Rapport’ refers to a sense of trust and familiarity built through repeated interaction. Studies of interactions between humans and AI agents (including robots and virtual AI agents) show that users receive psychological benefits when sharing information with AI agents similar to the benefits of sharing with human companions, or having pets in the household, including reduced negative mood and a sense of rapport (Akiyoshi et al., 2021; Gratch & Lucas, 2021; Holthöwer and van Doorn (2022); Luo et al., 2022; Laban & Cross, 2023; Laban et al., 2023; Alwafi & Fakieh, 2024). People also tend to become more trusting and more comfortable sharing with AI as they become more familiar with them, and these positive feelings encourage reciprocity (Laban et al., 2024; Manzini et al., 2024). While these effects erode over time with simpler social robots (Croes & Antheunis, 2020), the sophistication of modern AI chatbots supports more complex and prolonged relationship patterns.

Importantly, these tendencies are encouraged through intentional design. Corporations and other producers are incentivised to design AI anthropomorphically to foster these enduring and trusting relationships with users, as this increases user engagement (Balakrishnan & Dwivedi, 2021; Cominelli et al., 2021; Pizzi et al., 2023; Rheu et al., 2020; Zhang & Rau, 2023). Producers are even incentivised to create agents that are anthropomorphic in progressively advanced ways, such having a voice or facial expressions (Chen et al., 2021; Li & Sung, 2021). We expect that as AI agents become increasingly anthropomorphic, people will often form closer relationships characterized by positive feelings and disclose more information with these agents in turn.

In addition to anthropomorphic features, dark patterns may also be exploited by developers or consumer platforms in order to elicit personal information from users. Dark patterns may even overlap, interact with, or lurk within anthropomorphic features. For example, chatbots or personal assistants may employ human-like voices to encourage user trust while also subtly manipulating users to reveal specific kinds of information about themselves, such as consumer preferences (Song & Yun, 2025). They may exploit relational norms by exaggerating their needs or the benefits they provide to users in order to encourage increased disclosure or other user behaviors (Earp et al., 2025). The increased disclosure achieve through dark patterns further interacts with other properties of AI agents, such as difficult deletion (if the information is encoded in large AI models) or third-party access, to increase the downstream dissemination of personal information.

These patterns of human psychology, in conjunction with properties of AI agents, suggest multiple mechanisms that may increase people’s tendencies to disclose information. Just as importantly, the features of AI agents also appear to be poised to exploit that indiscretion. AI agents will have increased capacity to acquire and infer information about us, and third-parties may have unfettered access to that information.

On the other hand, some empirical results point against increased disclosure. In particular, in some contexts, people exhibit significant discomfort when interacting with AI agents or social robots, which can sometimes inhibit information disclosure (Bartneck et al., 2010; Berridge et al., 2023; Lutz et al., 2019). One hypothesized mechanism to explain discomfort is an ‘uncanny valley’ effect, wherein human users feel discomfort because the robot occupies an unfamiliar and uncanny middle ground between being fully human versus being not human at all (Mori et al., 2012; Pickard & Roster, 2020). To the extent that this hypothesis is true, we might expect that if robots and AI agents become sufficiently anthropomorphic, the uncanny valley effect may shrink or disappear. The effect may also diminish as human users become more familiar with interacting with robots or AI agents over time. Indeed, some evidence suggests that disclosure and comfort increase as human users have a prolonged course of interaction with robots (Ciechanowski et al., 2019; Kim et al., 2024; Pentina et al., 2023). An alternative proposed mechanism is inhibition due to negative emotions such as fear, frustration, or privacy worries (Hornung & Smolnik, 2022; Lutz & Tamò-Larrieux, 2021), which may not decrease with time or familiarity.

The apparent discrepancy between increased disclosure, as may be due to reciprocity or positive social feelings, and decreased disclosure, such as may be due to discomfort, is important to note. The discrepancy suggests that a thorough understanding of human-AI relational psychology is crucial for understanding and predicting the privacy impact of human-AI interactions, though more research is needed in order to better understand these effects. The discrepancy also suggests that, while interacting with AI agents, different users may respond in different ways and individual users may respond differently over time. Thus, while there is reason to worry about the risks of increased information disclosure, current predictions must be tentative and provisional.

As a final note, we wish to emphasize that even though the relationships between humans and AI agents may not be genuine, the research shows that relationship-like factors between humans and AI agents can affect how much information we disclose to them. So, the complaint that AI agents cannot form ‘real’ relationships does not undermine the relevance of human-AI relational psychology for understanding privacy.

## 4 Descriptive changes to privacy

So far, we’ve summarized philosophical theories of privacy, as well as several features of AI agents and human psychology that are relevant to privacy. In this section, we discuss how human-AI interactions may impact the descriptive facts about privacy–e.g., how information will flow in new ways as a result of these interactions. We structure this section according to the three main theories of privacy, using each approach to highlight changes to the privacy landscape. In the next section, we turn to a normative assessment by showing how these changes may promote or frustrate the values of privacy as described in section 2 and as summarized in Table 1.

### 4.1 Changes to access

As discussed in 2a, access theories of privacy highlight three ways that the acquisition of personal information by AI agents may in principle diminish privacy: direct strong access, indirect strong access, or weak access. Recall that weak access is the mere acquisition of information (including by, e.g., a webtracking bot) whereas strong access involves an observer (such as a human or possibly an AI agent). If AI agents are capable of strong access, then they can directly diminish privacy and its final values. Whether or not they are capable of strong access, they can indirectly diminish privacy and its final values by enabling strong access by humans (indirect strong access). If only weak access occurs, AI agents can still affect the instrumental values of privacy, such as when personal information is automatically used to screen job applications. Thus, while strong access marks a reduction in privacy, weak access only involves hypothetical risks that may be inflicted by another party.

When personal information is acquired by an AI agent, another person may ultimately access that information. AI agents have increased access to personal information by eliciting willing disclosure via anthropomorphic features or dark patterns, or by their sophisticated informational capacities. Because of the difficulty of deleting the information they acquire, their unpredictable behavior, and third-party access, AI agents may indirectly contribute to strong access by other humans, exposing the information they acquire to downstream privacy risks. Crucially, on the access conception of privacy, even willing disclosure of personal information diminishes privacy if it is accessed.

It’s clear that the acquisition of information by another person counts as strong access, but it’s less clear whether the acquisition of information by an AI agent itself counts as strong access. To answer this question, we need a general theory of the ‘grounds’ of the capacity for strong access.

We don’t have space to examine this question in detail, but we briefly mention two views about the grounds of strong access. These two views correspond to two notions of *awareness*. One sense of awareness refers to knowing and understanding information, such as when someone says, “I’m aware that it’s Tuesday.” The other sense of awareness refers to experiential or conscious awareness, such as when someone says, “I’m aware of my surroundings.” These two notions correspond roughly to a distinction in the philosophy of mind between *access consciousness* and *phenomenal consciousness* respectively (Block, 1995; cf. Hildt, 2022, Evers et al., 2025). In our view, it’s plausible that at least one of these forms of awareness is sufficient for grounding strong access.

A version of the view that understanding grounds strong access has been defended in the privacy literature. According to this view, an observer is capable of strong access if it is capable of *semantic understanding* of the information in question (Macnish, 2020; Soifer & Elliott, 2014). A closely related view is that strong access consists in possessing knowledge about personal information (Parent, 1983), but we focus here on the semantic understanding view. The semantic understanding view is plausible because semantic understanding and interpretation are closely connected: what it takes to form an interpretation of someone plausibly just is the capacity for semantic understanding of information about them. So, this account of the capacity for strong access aligns with what is required to affect final values of privacy. If this view is correct, then AI agents may be capable of access relatively soon, even before they develop any form of phenomenal consciousness (Verschure, 2016).

The second view holds that phenomenal consciousness is sufficient for strong access, where a capacity for phenomenal consciousness is a capacity for subjective experience. One reason in favor of this view is that, arguably, an observer must be phenomenally conscious in order for their acquisition or understanding of information to be valuable or disvaluable. If this view is correct, then it is perhaps less likely but still not impossible that AI agents will be capable of strong access soon (see Butlin et al., 2023 and Chalmers, 2023 for arguments that AI systems may have phenomenal consciousness in the near future).

If AI agents are capable of strong access, then the privacy impact of their information acquisition is compounded: not only can third-party humans diminish privacy, but the AI agents themselves can directly diminish privacy. This possibility is especially concerning because AI agents may be ubiquitous in the near future, thereby drastically shaping how individuals present and are interpreted or understood by others. Virtually every action may one day be observed and interpreted by an aware AI agent, diminishing the times and places in which individuals are not strongly accessed. One’s behavior may then nearly always be public and under scrutiny, in some sense, and our ability to be alone may evaporate.

On the other hand, it is also worth noting that if AI agents are not capable of strong access, and if the personal information they acquire can be firewalled or secured, then it is possible that AI agents could reduce strong access in some ways. For example, an AI psychotherapist or medical chatbot could provide “accessless” care or advice, whereas a human therapist cannot.

### 4.2 Changes to control

Several features of AI agents may affect the control that subjects have over their information. As already discussed at length, AI agents may elicit information through willing disclosure due to their anthropomorphic features. They may also elicit information through manipulation, especially via dark patterns. These mechanisms of elicitation—willing versus manipulated—differ from the point of view of control. Users exhibit a higher degree of control through willing disclosure in that they are wholly exercising their agency in the act of disclosure, whereas manipulation through dark patterns may undermine the agency of users to some extent. However, it is also important to notice that each mechanism can ultimately reduce people’s control over their information because they may not continue to exercise control after it has been revealed.

Beyond elicitation, the informational capacities of AI agents increase the personal information they can collect. For example, AI psychotherapists collect information about facial expressions and vocal tone and may even infer clients’ mental states from this information (Sawalha et al., 2022). If someone’s personal information can be observed or inferred by an uncontrollable AI agent, then they lack control over that information, too.

Here again we see an interaction between the properties of AI agents that produces special privacy risks, this time especially related to control. The properties of AI agents that increase the information they acquire introduce risks of dissemination due to the other properties of AI agents: their unpredictable behavior, the difficulty of deleting the information, and due to access by third parties. Given that modern AI systems are driven by complex statistical algorithms, they may act in unpredictable ways (Yampolskiy, 2024). If AI agents can disclose information to external systems or individuals, there is a risk that they may spontaneously share subjects’ information in ways that the subject could not have easily predicted, reducing the subjects’ control over their personal information. Similarly, if the third-parties that manage the AI agents (such as Google and its product Gemini) have access to the information acquired by those agents, then individuals may have little control over how their information is used.

The problem of deleting one’s information contributes to these risks. It can be especially difficult to delete information from AI systems that encode information in deep neural nets (Patil et al., 2023). Existing regulations enact a right to deletion of personal information (Regulation EU 2016), which helps to preserve people’s control over their information. However, these regulations do not straightforwardly apply to information encoded within AI via machine learning algorithms. So, people do not have a straightforward regulatory mechanism for deleting that information. It is also worth noting that due to the scale of their deployment, large AI models pose threats to group privacy by inferring information about groups even from data sets that do not include personally identifying information. We have set aside issues of group privacy in this framework, but the issue merits further attention (Floridi, 2016; Mittelstadt, 2017).

### 4.3 Changes to contextual integrity

The contextual integrity theory of privacy highlights other ways that human-AI interactions may affect the flow of information within contexts. When information flow changes in a given context, it may violate contextual information norms, thereby transgressing contextual integrity. We discuss four such changes here: AI agents may collect new types of information, information may be transmitted to or accessed by third-parties, AI systems may be new kinds of information receivers, and AI agents may abide by new transmission rules. Each of these changes is influenced by features of AI agents and human psychology already discussed.

First, when new kinds of information are collected in a context, that changes the flow of information in that context. Imagine, to demonstrate the point, that a doctor was able to read patients’ minds. Then, in the healthcare context in which that doctor was present, the doctor would be able to acquire new types of information about patients beyond what is standard in the healthcare context. The contextual integrity theory diagnoses this change in information flow as a diminishment of privacy that may have contextually significant ramifications. In particular, we might worry that a norm of healthcare information, such as that patient information is willingly disclosed, has been breached. The informational capacities of AI agents, in superseding the observational capacities of humans, may affect contextual integrity similar to how a doctor’s mindreading abilities would affect contextual integrity. The theory of contextual integrity flags such changes as diminishments of privacy, which must be assessed for how they promote or frustrate contextual and non-contextual ends.

Second, beyond having new informational capacities, AI agents may also transmit information to third-parties in contextually transgressive ways. OpenAI and Google are not currently receiving information about subjects’ healthcare, but AI agents could change that. The contextual integrity theory diagnoses these changes as potential diminishments of privacy, too.

Third, AI agents themselves may *be* new kinds of actors, whether as information receivers or senders—a change made possible because they have some anthropomorphic features. From the point of view of contextual integrity, the capacities and contextual roles of an actor determine what kind of actors they are. Because AI agents will have inhuman capacities or occupy contextual roles in new ways, they will be new kinds of actors. As an example, an AI psychotherapist may lack the ability to empathize with patients as a human psychotherapist could, altering the client-therapist relationship. The ability of a psychotherapist to empathize with a patient may even be integral to the patient’s reasons for sharing information. Then, sharing information with a non-empathetic AI psychotherapist diverges from the contextual norm. The contextual integrity theory diagnoses the presence of new kinds of actors as a potential breach of the norms of information flow.

Fourth, AI agents may introduce new operative transmission principles in a given context. Some of the ways they may do this have already been discussed, such as if they share information with a third-party owner of the AI agent platform or if information cannot be deleted from a model in a standard way. There are many possible ways that AI agents will change the operative transmission principles, and it is difficult to predict in advance the full range of ways that AI agents may introduce such changes. Indeed, people who interact with AI agents will also have difficulty predicting how the agents may share information. We will consider just one more example to demonstrate this point.

Consider again the context of mental health therapy. While the generally accepted transmission principle in the context of mental health therapy is confidentiality of the client’s information, there are special circumstances where confidentiality should be breached. In particular, if the client reveals that they pose a significant danger to themselves or others, then it is appropriate for the therapist to breach confidentiality in order to prevent harm to the client or to others. These rules and conditions are baked into the therapeutic context. If AI psychotherapists do not abide by the subtleties of conditional confidentiality, then the operative transmission principles diverge from what is standard. Then, contextual integrity flags the divergence as potentially problematic.

## 5 Evaluating the changes to privacy

**Fig. 1:**
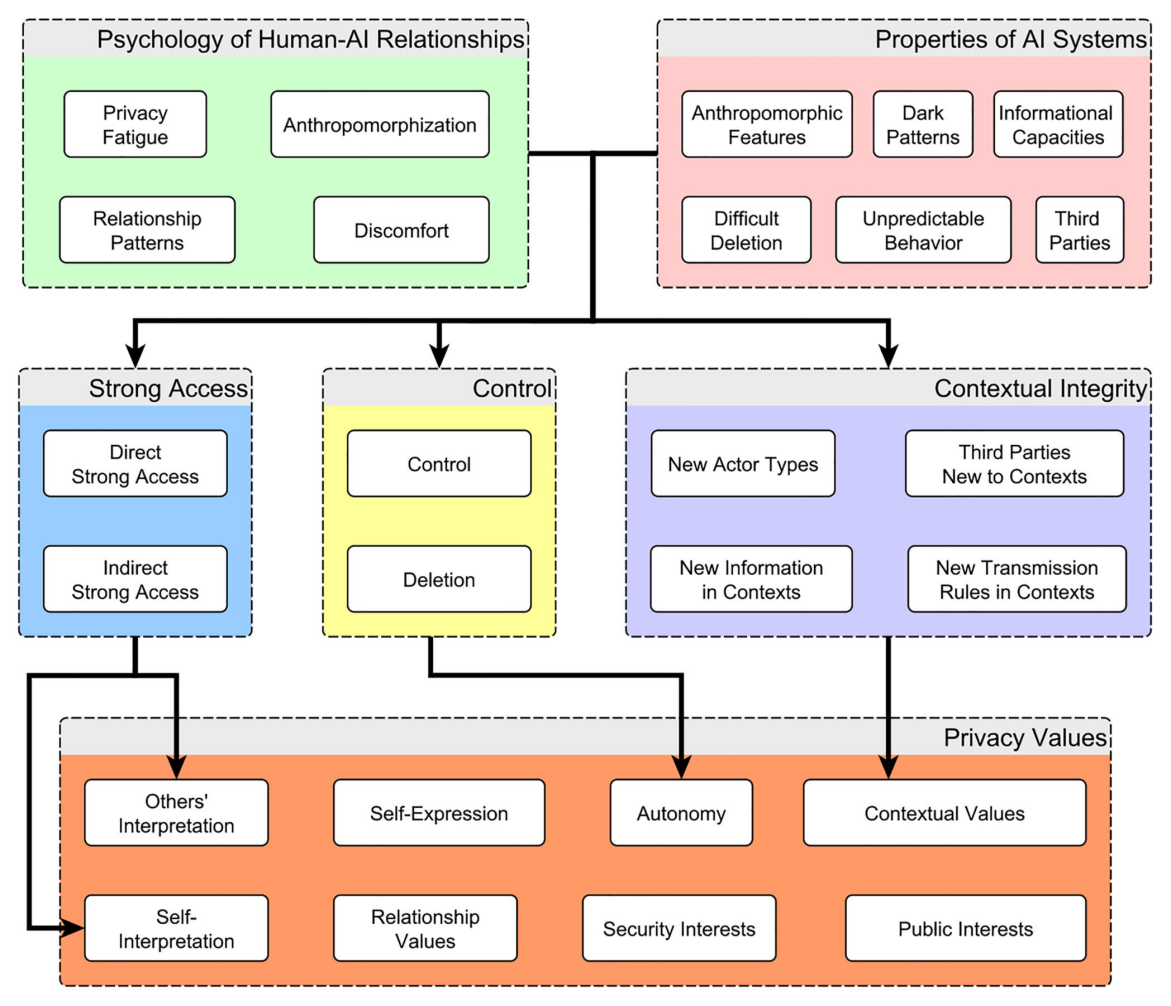
The Structure of the Framework

We have described how AI agents may change the landscape of privacy, and we now turn to normatively assessing the values and disvalues that may follow as a result. Before proceeding, it is helpful to have in mind the structure of the framework we have been developing, depicted in Fig. 1. The figure summarizes how the psychology of human-AI relationships and the features of AI agents jointly affect the features of the privacy landscape (grouped according to three theories of privacy) which in turn affect eight valuable features of privacy, taken from Table 1 in section 2. The values of privacy are affected in complex ways by access, control, and contextual integrity, but drawing an arrow for every causal relationship is too convoluted to depict. When a particular conception connects especially closely to a particular value (e.g., strong access to interpretation, control to autonomy, contextual integrity to contextual values), an arrow makes this connection explicit. When no arrows are drawn to a value of privacy, it should be understood that each conceptualization of privacy is relevant to that value to some extent. Additionally, we focus primarily on potential negative effects, though some potential benefits are mentioned.

In the remainder of our discussion, we will assess how the descriptive changes to privacy bear on the eight values of privacy shown at the bottom of Fig. 1.

### 5.1 Others’ interpretation

When others have strong access to an individual’s personal information, that can affect how they interpret that individual. People have interests in how they present and are interpreted by others, and so strong access bears on the value of others’ interpretation for those individuals.

In principle, the value of others’ interpretation of an individual can be positively or negatively affected by strong access. If the strong access of someone’s information promotes how they want to be interpreted, then that is positively valuable for that individual. If, on the other hand, that information promotes an undesired interpretation, such as by casting the individual in a bad light, that is disvaluable for them.

If AI agents are capable of direct strong access, or if they indirectly enable strong access by other people, then they can directly or indirectly affect how an individual is interpreted. AI agents may have distinctive effects on interpretation due to their peculiar features, such as their informational capacities. Because they are able to collect and infer new kinds of information about us, they may enable interpretations of us based on those new kinds of information. Whereas people have some ability to notice subtle physiological changes or make sophisticated inferences to “read between the lines” of what is spoken, AI agents in the future may exceed these abilities.

Direct or indirect access that is enabled by AI agents may be valuable or disvaluable not simply in relation to the desired interpretations, but also because it can affect what Elliott and Soifer (2022) call *epistemic privilege*—the privilege frequently enjoyed by individuals when they know more about themselves than others do. While Elliott and Soifer (2022) describe downstream benefits of this privilege (such as may be relevant to relationship formation), it’s worth observing that this privilege may be valuable to someone as an end in itself or because it avoids discomfort. Thus, by contributing to how individuals are interpreted or by impacting their epistemic privilege, AI agents pose distinctive risks of directly diminishing privacy.

If AI agents are not capable of direct access, there are potential benefits. As with the example of “accessless” therapy mentioned earlier, individuals may be able to share personal information without fear of judgment. This opportunity could have significant benefits, such as by facilitating mental healthcare for those who would otherwise be unwilling (Fiske et al., 2019).

### 5.2 Self-interpretation

How others interpret a person is not the only sort of interpretation that matters. The narrative conception we have of ourselves–how we interpret and make sense of our actions, traits, and course of life in relation to the world and others around us–is often important to us. That is, it can be important how we interpret ourselves (Shoemaker, 2010).

Is self-interpretation a matter of privacy? Arguably, yes. When one’s information is accessed or interpreted by others in a certain way, that can encourage or force a certain self-interpretation. How we understand ourselves is often conditioned by how we see ourselves through others’ eyes, and so how those others interpret us can make a difference to our self-interpretation. The value for us of our self-interpretation thus becomes an additional value of privacy, closely tied to how we are accessed and interpreted by others.

By enabling and increasing direct or indirect access, AI agents may force individuals to confront possible interpretations of their traits and actions that they would otherwise be able to avoid. This confrontation could have benefits, such as by increasing self-awareness, but it could also have serious costs, such as by encouraging selfcriticism or unpleasant forms of self-consciousness such as shame or embarrassment.

### 5.3 Self-expression

The possibility of direct or indirect access can also affect individual’s willingness to express themselves or to pursue activities as they wish. These effects can come about directly via the imposing presence of AI agents, or indirectly by raising the salience for people of harms that might be inflicted by other people or institutions. The possibility of weak access, such as may enable institutional harms inflicted on an individual (e.g., being disadvantaged or targetted based on personal information), can affect such self-expression such as by ‘chilling’ or deterring people from exercising their freedoms of expression. These ‘chilling effects’ can impede an individual’s desired form of life and deprive them of many goods (Büchi et al., 2022; Penney, 2022).

AI agents, in enabling and increasing both strong and weak access, may exacerbate chilling effects. They may also introduce chilling effects into new domains, such as in one’s own home or workplace, where before one might have remained unobserved and unaccessed. The increased encroachment of AI agents in our lives may thus spread chilling effects, further stifling free self-expression or confining it to ever-shrinking corners of the world.

We also note the possibility that AI agents may provide benefits with respect to self-expression, such as by affording individuals an audience (real or pretend) they might otherwise lack. Having such an audience could be motivating or otherwise beneficial for some people, especially those who are afraid in particular of human audiences (Lucas et al., 2014).

### 5.4 Relationship values

The privacy impact of human-AI interactions may affect relationship values in complex ways that we cannot thoroughly canvass here. Increased access and reduced control, as we have discussed, may make it more difficult for individuals to manage the flow of information in ways that promote the formation and maintenance of relationships as they desire. If my personal AI assistant discloses sensitive information to your personal AI assistant, then that may indirectly diminish my privacy and set back my ability to maintain the desired relationship with you.

On the other hand, if AI agents are adept at managing personal information in appropriate ways, then they could in theory improve or at least preserve our control over our relationships. For example, personal AI assistants could negotiate when and where two people should meet without later revealing sensitive information to the humans themselves. Achieving that sort of social adeptness is a difficult project in value alignment that is currently underway (Gabriel et al., 2024; Ghalebikesabi et al., 2024).

### 5.5 Autonomy

We have discussed how interactions with AI agents may reduce individuals’ control over their information, which then reduces their control over important aspects of their lives and hence reduces their autonomy. Just how drastically might autonomy be curtailed, and how bad would that be? Already, webtracking and dark patterns in digital technologies result in institutions shaping individuals’ environments and even manipulating those individuals in ways they do not request or control (Véliz, 2020). Interactions with AI agents may exacerbate these detriments to autonomy due to their informational capacities, the access of that information by third-parties, and our greater tendencies to disclose information to those agents. Because AI agents act in unpredictable ways and are driven by complex and opaque algorithms, it will not be easy for individuals to execute their plans or values through these agents. So, control and hence autonomy could be severely curtailed.

In a world increasingly shaped by the interests and machinations of large corporations and other institutions, as well as oppressive and intolerant social regimes, it’s hard to overstate the significance of further setbacks to individuals’ autonomy. Social, political, and economic disempowerment is an important privacy concern (cf. Valentini, 2022) and a concern of AI development in general (cf. Bales, 2024). We expect the intersection of these concerns to be especially serious.

On the other hand, it is also possible that AI agents designed to align with individual interests and wishes could promote autonomy, enabling individuals to exercise control over choices even when they cannot be personally present, including for relationship goals (Danaher & Nyholm, 2024; Voinea et al., 2024). Ensuring that these technical developments preserve and promote the values of privacy in general is an ongoing issue to address.

### 5.6 Security interests

Many of the effects of human-AI interactions discussed so far will also bear on individuals’ security interests. Recall that a security interest (see Table 1) is an interest in avoiding harm that could be inflicted by an abuse of their personal information, such as in cases of reprisal for one’s political views or actions. To the extent that AI agents increase weak access or diminish control, they increase downstream threats to individuals’ security interests.

### 5.7 Contextual values

The proliferation of AI agents in a variety of social contexts promises to change the flow of information in a multitude of ways. In each case, the theory of contextual integrity raises a red flag, calling for an overall evaluation of the change to assess whether contextual integrity recommends against it. Faithfully carrying out this assessment according to the augmented decision heuristic of Nissenbaum (2010) is a delicate and demanding enterprise—one that ought to be performed in detail on a case-by-case basis. We will merely sketch an example assessment.

Consider again the case of an AI psychotherapist. In a context where a client displays some evidence that they may harm themselves or others, an AI therapist may be designed to report this incident to other parties with greater or lesser sensitivity than a human therapist would. If an AI therapist is overly sensitive to such risks, then it may breach confidentiality too often, resulting in unnecessary violations of trust and confidentiality. If the AI therapist is not sensitive enough, then it may fail to warn others of harm when a human therapist may have done better. Designing an AI system to be appropriately sensitive is presumably a very difficult task, requiring sensitivity to subtle communicative cues and detailed knowledge of human behavior in general and of the client in particular. Mistakes of either variety are serious, so the risk of harm is high.

The theory of contextual integrity emphasizes the importance of contextual ends in assessing these information transmission practices. A central end in the context of psychotherapy is the promotion of patient wellbeing. Mistakes of either variety pose risks to patient wellbeing, but there are other important factors too. A patient may be aware of the fact that an AI therapist could have a different sensitivity to the threat of harm than a human therapist would. The patient may even have a hard time judging what that sensitivity is, given that AI systems often exhibit unpredictable behavior governed by opaque, complex algorithms. As a result, patients may be less comfortable sharing sensitive information if they feel they cannot trust the AI therapist to make a wise decision. This factor may also be a detriment to patient outcomes.

Contextual ends are not the only ends that matter, of course. Other factors such as the public costs and benefits of the practice are relevant, making an overall assessment complex.

### 5.8 Public interest

Privacy is of value to the public interest in many ways. It can encourage free participation in democratic institutions where individuals do not fear reprisal. It can also promote safeguards that allow people to explore and develop themselves outside of the public eye, leading to cultural richness or a diversity of skills that may contribute to a healthy society. For these reasons, chilling effects, as discussed regarding the value of self-expression, and individual’s security interests each feed into values that are of public interest too.

Due to the complexity of factors, it is difficult to predict how a proliferation of AI agents may affect public interest on balance. Even so, it is worth dwelling on the potential costs. If people are constantly acting and interacting in the presence of or with AI agents, then our behaviors and mental states will be recorded and potentially preserved to a far greater extent than they currently are. If the information is especially difficult to delete, or if third-parties have access to that information, then powerful institutions will be in a position of power over individuals, greatly increasing the potential public costs of chilling effects and individual harms.

## 6 Conclusion

We have drawn from theories of privacy and its value, as well as current evidence about the properties of AI agents and human-AI relational psychology, to sketch a framework for assessing the privacy-ethical impact of AI agents. We first developed a broad conception of information privacy as concerning the actual and potential flow of personal information, which integrates conceptions of privacy in terms of access, control, and contextual integrity. This ecumenical perspective has the advantage of capturing the plurality of values that privacy serves to promote.

We then discussed six properties of AI agents and four patterns of human relational psychology that we believe are important factors in determining the privacy impacts of AI agents. The most distinctive property of AI agents—their anthropomorphic features—figures centrally in this discussion because it brings human relational psychology into the fold. The explicit incorporation of relational psychology is a key innovation of the framework we provide. After highlighting how these properties and psychological patterns combine to influence the flow of information to and from AI agents, we then identify how these factors change privacy according to our tripartite conception. Our analysis reveals that AI agents may threaten privacy directly by diminishing privacy via the acquisition of personal information and indirectly by increasing hypothetical risks imposed by other people or institutions.

Finally, we provided a preliminary assessment of how AI agents may bear on eight distinct values of privacy, informed by the earlier tripartite analysis. While our assessment is necessarily incomplete due to the complex and dynamic introduction of AI agents into society, we hope that our framework will prove useful for future, more complete assessments. We also hope that future research on human-AI relational psychology will continue to improve our understanding of the privacy impact of human-AI relationships.

## Figures and Tables

**Table 1:** The Values of Privacy

Value	Definition	Beneficiaries	Examples
Other-interpretation	How the information subject is understood and interpreted by others who access their information	Subject	Being judged by others; one’s self-presentation
Self-interpretation	How the information subject interprets and feels about themselves in light of others’ access of their information	Subject	Shame, embarrassment, comfort, self-esteem, and narrative costs or benefits
Self-expression	How (potential) access of a subject’s information affects their dispositions to express themselves	Subject & Society	Inhibition or discouragement of self-expression
Relationship values	How (potential) access of a subject’s information affects the maintenance and formation of their relationships	Subjects & Society	Value of associations for individuals and society
Autonomy	The autonomy a subject possesses over their life in virtue of having control over their information	Subject	Autonomy as valuable in itself; instrumental value of (e.g.) avoiding discrimination
Security interests	A subject’s interests in avoiding harm from institutions that could be inflicted if their information is accessed	Subject	Avoiding oppression or social approbation
Contextual values	Various contextual values that are promoted or protected by contextual integrity	Subject & Society	Patient healthcare outcomes; student educational outcomes
Public interest	Public goods that result from adherence to privacy norms of various kinds	Society	Competent electorate; benefits that arise from cultural diversity

## Data Availability

Not applicable

## Box 1 Elements of the Theory of Contextual Integrity

(1) Data subject: The person that the information is about
(2) Sender: The person or system in a position to send the subject’s information
(3) Receiver: The person or system in a position to receive the subject’s information
(4) Information Type: The type of information about the data subject that may be transmitted
(5) Transmission Rule: The conventional rule that governs the flow of the data subject’s information between senders and receivers in a context
(6) Context: An established social or institutional setting, characterized by established roles, relationships, and norms. Privacy norms inhere in contexts and are characterized by the parameters in (1)-(5).

## Box 2

(1) Anthropomorphic Features: AI agents have anthropomorphic features such as the language fluency and conversational ease, which invites anthropomorphization by people who use them.
(2) Dark Patterns: Many consumer-facing digital technologies are designed with ‘dark patterns’ that manipulate user behavior, such as to limit or shape user choices. AI agents may exhibit dark patterns that manipulate users into disclosing information (Song & Yun, 2025; Waldman, 2020).
(3) Informational Capacities: AI systems will increasingly have greater observational and inferential capacities than other information technologies, increasing the amount and types of information that they can acquire, learn, and deploy (Martin & Zimmermann, 2024).
(4) Difficult Deletion: The information encoded in AI systems built with deep neural networks is difficult to delete, reducing the control that subjects have over information that may be included in training data (Patil et al., 2023).
(5) Unpredictable Behavior: AI agents exhibit complex behavior that is in many ways harder to examine and predict than other information technologies (Yampolskiy, 2024).
(6) Third Parties: AI agents are often managed by third-parties, such as the technology companies that develop them. These third-parties may have access to information that the AI systems acquire (Casper Ferm et al., 2022).

## Box 3

(1) Privacy Fatigue: People tend to become emotionally and/or cognitively overwhelmed by the demands of managing their personal privacy, especially when information flow is complex or opaque (e.g., social media, AI agents like ChatGPT).
(2) Anthropomorphization: People tend to anthropomorphize objects and interact with them in quasisocial ways, especially when those objects have anthropomorphic features.
(3) Relationship-patterns: As people interact with AI agents, they sometimes respond as though they were in a relationship, developing a sense of rapport, reciprocity, and other quasi-social feelings and patterns, affecting the information they may disclose.
(4) Discomfort: Discomfort and inhibition may sometimes decrease the amount of information that humans disclose to AI agents, depending on social contexts and the features of the AI agents.
